# Food Access and Perceptions of the Community and Household Food Environment as Correlates of Fruit and Vegetable Intake among Rural Seniors

**DOI:** 10.1186/1471-2318-10-32

**Published:** 2010-06-02

**Authors:** Joseph R Sharkey, Cassandra M Johnson, Wesley R Dean

**Affiliations:** 1 Program for Research in Nutrition and Health Disparities, School of Rural Public Health, Texas A&M Health Science Center, MS 1266, College Station, TX 77843-1266, USA; 2 Texas Healthy Aging Research Network (TxHAN) Collaborating Center, Center for Community Health Development, School of Rural Public Health, Texas A&M Health Science Center, MS 1266, College Station, TX 77843-1266, USA

## Abstract

**Background:**

Although the importance of fruit and vegetable consumption to health has been well established, few studies have focused on access to fruits and vegetables in rural areas; even fewer examined the relationship between food access and fruit and vegetable consumption among seniors.

**Methods:**

To examine the spatial challenges to good nutrition faced by seniors who reside in rural areas and how spatial access influences fruit and vegetable intake.

A cross-sectional analysis using data from the 2006 Brazos Valley Health Assessment (mailsurvey) for 582 rural seniors (60-90 years), who were recruited by random digit dialing; food store data from the 2006-2007 Brazos Valley Food Environment Project that used ground-truthed methods to identify, geocode, and inventory fruit and vegetables in all food stores.

**Results:**

Few of the BVHA seniors consumed the recommended intakes of fruits or vegetables; women consumed more servings of fruit (1.49 ± 0.05 vs. 1.29 ± 0.07, *p *= 0.02), similar servings of vegetables (2.18 ± 0.04 vs. 2.09 ± 0.07, *p *= 0.28), and more combined fruit and vegetables (3.67 ± 0.08 vs. 3.38 ± 0.12, *p *= 0.04) than men. The median distances to fresh fruit and vegetables were 5.5 miles and 6.4 miles, respectively. When canned and frozen fruit and vegetables were included in the measurement of overall fruit or vegetables, the median distance for a good selection of fruit or vegetables decreased to 3.4 miles for overall fruit and 3.2 miles for overall vegetables. Almost 14% reported that food supplies did not last and there was not enough money to buy more. Our analyses revealed that objective and perceived measures of food store access - increased distance to the nearest supermarket, food store with a good variety of fresh and processed fruit, or food store with a good variety of fresh and processed vegetables - were associated with decreased daily consumption of fruit, vegetables, and combined fruit and vegetables, after controlling for the influence of individual characteristics and perceptions of community and home food resources.

**Conclusions:**

Findings suggest that interventions designed to increase fruit and vegetable consumption among rural seniors should consider strategies to ameliorate differential access to healthy food due to food store distance.

## Background

The percentage of older adults with nutrition-related health conditions, such as obesity, diabetes, cardiovascular disease, and some cancers has been increasing [1]. Healthy eating, such as the consumption of fruit and vegetables is now recognized as one modifiable determinant for the prevention and management of chronic health conditions; and is targeted in national recommendations [2-7]. However, the current focus on fruit and vegetables is limited to fresh fruit and vegetables [8-17], which ignores dietary recommendations and the nutrient benefits of canned and frozen fruit and vegetables [3,18].

Personal and environmental characteristics result in differential access to health resources and serve as either barriers or enhancements to healthy eating, especially in rural areas [19,20]. Without access to a supermarket, vulnerable populations like older adults may struggle to obtain the food needed for a healthy diet and face an increased risk of diet-related chronic disease [21-23]. Previous studies have examined personal or individual factors such as gender, income, and education and how these factors affect health and nutrition [24-26], while more recent studies have considered the effect of aspects of the built environment on diet [9,27-31]. Urban populations have been the primary target for this latter research focused on identifying environmental barriers to healthy eating in the U.S. [32-36] and elsewhere [37-43]. However, few studies examined food access and shopping among older adults [44-46]. Further, only a limited number of studies focused on environmental factors and their influence on access to food among rural populations [47-53], and none were found examining environmental barriers to healthy foods among older adults in rural areas.

Physical access is a major problem for people without cars, the elderly, people on low incomes, and residents in rural areas [51,54]. There is strong evidence that residents of rural areas are affected by poor access to supermarkets and healthy foods [49-51,55-58]. However, little is known about the spatial challenges to good nutrition faced by seniors who reside in rural areas and how spatial access influences fruit and vegetable intake. Nutritional disparities faced by rural seniors make understanding access to a variety of fruits and vegetables critical. The aims of this study are to (a) depict potential spatial access from rural neighborhoods to a mix of retail food stores that market fruit and/or vegetables; (b) describe individual and neighborhood characteristics, spatial access to food resources, and fruit and/or vegetable intake; and (c) examine the associations among individual and neighborhood characteristics, perceived and objective measures of food access, and fruit and/or vegetable intake of rural seniors. We hypothesized that rural seniors who reside at a greater distance from food stores would report lower fruit and/or vegetable intake.

## Methods

### Sample and Study Design

**Figure 1:**
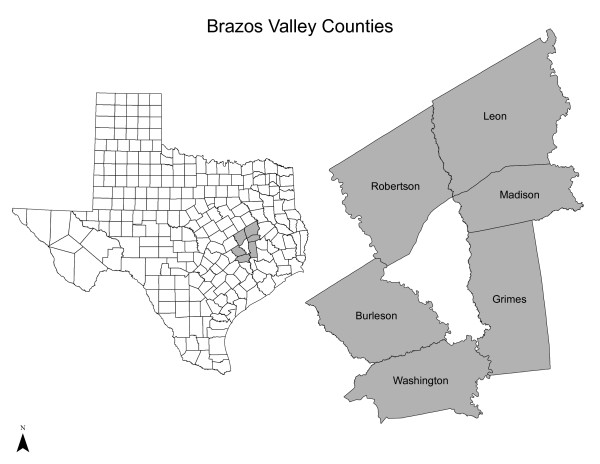
**Map of Texas and rural Brazos Valley Counties**.

We used data from the 2006 Brazos Valley Health Assessment (BVHA), the 2006-2007 Brazos Valley Food Environment Project (BVFEP), and the decennial 2000 U.S. Census Summary File 3 (SF-3) for a 6-county rural area (see Figure 1). The rural study area included 101 census block groups (CBG), land area of approximately 4,500 square miles, and population of more than 119,650 people [51,59]. Regular public transportation services, such as fixed route, commuter, or taxi services, were not available in the study area [60,61]. Data for the BVHA were collected from 663 rural seniors (age ≥60 years) who were recruited into a large community assessment through random digit dialing and follow-up mail survey; detailed methodology has been described elsewhere [62]. The analytic sample included rural seniors with residential addresses and complete nutrition data (*n *= 582); all participants were geocoded to their residence. In the BVHA, respondents were asked about daily intakes of fruit and/or vegetables, availability and perception of community retail food resources, household food resources, and demographic characteristics. The BVHA and BVFEP were approved by the Institutional Review Board at Texas A&M University.

### Measures

#### Fruit and vegetable intake

Fruit and vegetable intakes were separately measured by a validated, self-reported two-item screener [63,64]. One item asked participants to report the number of servings of fruit (1/2 cup of fruit or ¾ cup fruit juice) usually consumed each day; the second item targeted the number of servings of vegetables (1/2 cup cooked or 1 cup raw) consumed daily. In addition to separate intakes of fruit and vegetables, a combined fruit and vegetable intake variable was calculated as the total of both fruit and vegetable intakes. *Community retail food resources*. The perceived adequacy of community food resources was assessed using a 5-point Likert scale (e.g., 1 = strongly agree to 5 = strongly disagree) for three items about the local community: 1) little variety in types of foods that can be purchased; 2) few grocery stores or supermarkets; and 3) food prices are high. Responses were collapsed into binary variables, based on strongly agree/agree vs. other responses. Perceptions related to the store where most of the groceries were purchased were assessed on a 5-point Likert scale (e.g., 1 = excellent 5 = poor) using three questions: 1) how would you rate the variety of fruits and vegetables at this store; 2) how would you rate the freshness of fruits and vegetables; and 3) how would you rate the price of fruits and vegetables? Binary variables were constructed as fair/poor vs. all others. *Household food resources*. Adequacy of household food resources was assessed using three items. Two statements asked whether the following was often true, sometimes true, or never true in their household in the past month: 1) the food we bought last month didn't last and we didn't have enough money to buy more; and 2) we couldn't afford to eat balanced meals. Binary variables were constructed as often/sometimes true vs. never true. In the third question, participants were asked (yes or no) whether they and/or other adults in the household ever cut the size of meals or skipped meals in the last 12 months because there wasn't enough money to buy food.

*Sociodemographic characteristics *included age (range of 60-90 years), gender, race/ethnicity (all minority vs. non-Hispanic white), household income (≤100% Federal Poverty Level [FPL], 101%-199% FPL, and ≥200% FPL), years of education completed (low [< high school] vs. high [≥high school graduate]), marital status (married vs. not married), and household composition (lives alone vs. lives with others).

#### Objective measurement of fruit and vegetable availability

BVFEP data included the on-site identification and geocoding of all supercenters, supermarkets, grocery stores, convenience stores, dollar stores, mass merchandisers, and pharmacies; and completion of an observational survey by trained researchers of the availability and variety of fresh and processed (canned, frozen, and juice) fruits and vegetables in the 185 food stores that marketed some form of fruit or vegetable [51,55,65]. The availability of fruit and vegetables was separately determined from the presence and variety of fresh and processed fruits and vegetables [55,65]. Processed fruit and vegetables included canned (in natural juice or in light syrup), frozen (without added sugar or sauce), and 100% juice items [18]. Variety was defined as the number of different food items within a fruit or vegetable category (e.g., number of different fresh fruits). Based on overall fruit and vegetable scores, four variables were constructed for good availability of fresh fruit, fresh and processed fruit, fresh vegetables, and fresh and processed vegetables [65].

#### Residential neighborhood measures

Socioeconomic characteristics were extracted from the SF-3 for all 101 CBG in the rural study area to describe socioeconomic deprivation and population density [51]. Home addresses for all respondents were used to identify the neighborhood or CBG of residence. Based on the distribution of weighted socioeconomic deprivation scores, neighborhoods (CBG) were classified as being low, medium or high deprivation [51]. Population density was calculated as the number of persons/mi^2 ^in each CBG. Neighborhood deprivation and population density data were linked to each respondent through the residential CBG.

#### Potential spatial access to fruit and vegetables

Network distance was calculated with ESRI's Network Analysis extension in ArcInfo 9.2, which computed the distance along the road network to the geographic position measured in front of each food store. Separate network distances were calculated from the residential address of each BVHA senior respondent to the nearest corresponding supermarket, and food store (regardless of type) with a good selection of fresh fruit, fresh and processed fruit, fresh vegetables, or fresh and processed vegetables. The network distance to the nearest food store was calculated between paired point data (respondent address and nearest corresponding food store within the study area).

### Statistical analyses

Release 11 of Stata Statistical Software was used for all statistical analyses; *p *<0.05 was considered statistically significant. Descriptive statistics were estimated for sociodemographic characteristics; residential neighborhood; fruit and vegetable intake; distances from residence of each respondent to the nearest supermarket and nearest food store (regardless of type) that offered a good selection of fresh fruit, fresh and processed fruit, fresh vegetables, or fresh and processed vegetables; and community and household food resources. A 2-phase approach was used to examine the association of sociodemographic and neighborhood characteristics with fruit and/or vegetable intake. First, bivariate correlations between fruit and/or vegetable intake and sociodemographic characteristics, residential neighborhood, community retail food resources, and household food resources were estimated using Pearson's product-moment correlation. Second, multivariable linear regression models were individually fitted using backward elimination strategy to determine the relationship of sociodemographic characteristics, community and household food resources, and network distance to food stores with fruit and/or vegetable intake. Variables that were associated with fruit and/or vegetable intake (*p *< 0.10) were included in the multivariable models. Separate models were estimated for three outcome variables, with robust (White-corrected) SEs for heteroscedasticity of unknown form: fruit intake, vegetable intake, and combined fruit and vegetable intake. In model 1, all candidate variables from the bivariate analyses were simultaneously entered, with backward elimination of variables not statistically significant (*p *<0.05). Network distance to the nearest supermarket was added as an explanatory term. In model 2, distance to nearest supermarket was replaced with distance to the nearest food store with a good selection of fresh fruit or vegetables. In model 3, network distance to the nearest food store with a good selection of fresh and processed fruit or vegetables replaced distance to the nearest food store with a good selection of fresh fruit or vegetables s in model 2.

## Results

### Sample and neighborhood characteristics

Sample characteristics between the analytic sample of 582 seniors who completed all nutrition-related questions in the BVHA and the 663 rural seniors who returned surveys were not significantly different (data not shown). The mean age for the 582 BVHA respondents was almost 70 years; 68% were women; almost 65% were married; and 27% lived alone (Table 1). More than 33% of the seniors reported a household income below 200% of the Federal Poverty Level; women were more likely to report a poverty level income than men (20.4% vs. 9.1%, *p *< 0.001), as well as older seniors (*p *< 0.001). Almost one-fourth of respondents resided in a high deprivation neighborhood; the neighborhood population density was less than 14 persons/mi^2 ^for 27% of respondents. Few of the BVHA seniors consumed the recommended intakes of fruits or vegetables; women consumed more servings of fruit (1.49 ± 0.05 vs. 1.29 ± 0.07, *p *= 0.02), similar servings of vegetables (2.18 ± 0.04 vs. 2.09 ± 0.07, *p *= 0.28), and more combined fruit and vegetables (3.67 ± 0.08 vs. 3.38 ± 0.12, *p *= 0.04) than men.

**Table 1:** Characteristics of Rural Seniors in 2006 Brazos Valley Health Assessment (n = 582)

	% (*n*)	Mean ± SD
***Individual characteristics***		
Age, y		69.92 ± 6.91
Women	68.2 (397)	
Race/ethnicity		
Minority	14.4 (84)	
Household income		
≤ 100% FPL	17.0 (99)	
101-199% FPL	16.3 (95)	
Education		
Low (< High school)	13.1 (76)	
Marital status		
Married	64.8 (377)	
Household composition		
Lives alone	27.7 (161)	
***Residential Neighborhood***		
Deprivation, % (*n*)		
Low	29.6 (172)	
Medium	46.2 (269)	
High	24.2 (141)	
Population density (persons/mi^2^)		
Low (<14)	27.0 (157)	
Medium (14-127)	46.6 (271)	
High (>127)	26.5 (154)	
***Daily dietary intakes***		
Fruit		1.43 ± 0.98
Vegetables		2.15 ± 0.91
Combined fruit and vegetables		3.58 ± 1.60

SD = standard deviation

### Objective measures of potential food access

The rural food environment consisted of 186 food stores, including one supercenter, 11 supermarkets, 12 grocery stores, 141 convenience stores, 16 dollar stores, four mass merchandisers, and one pharmacy [51]. Fruit and vegetable data were collected in 185 food stores; one convenience store was excluded because of refusal for an in-store survey of food items [65]. Using network distance measures from each participant's residence to the nearest supermarket, the median distance was 8.7 miles (Table 2). Food stores, regardless of type, with a good selection of fresh and processed fruit and vegetables were identified from in-store food surveys [55]. The median distances to fresh fruit (5.5 miles) and fresh vegetables (6.4 miles) were greater than median distances to the nearest store with a good selection of fresh and processed fruit (3.4 miles) or vegetables (3.2 miles).

**Table 2:** Potential spatial access (in miles) from rural senior's residence to the nearest supermarket and good selection of fruit and vegetables (*n *= 582)

		Mean (SD)^a^	Median	IQR^b^
Supermarket		9.9 (9.2)	8.7	1.06 - 14.46
Fresh				
Fruit		6.1 (5.3)	5.5	0.87 - 9.65
Vegetables		6.7 (5.7)	6.4	0.97- 10.47
Fresh and processed^c^				
Fruit		4.4 (4.1)	3.4	0.58 - 7.62
Vegetables		4.2 (4.0)	3.2	0.65 - 6.89

^a ^SD = standard deviation

^b^IQR = interquartile range (first to third quartiles)

^c^Processed = canned, frozen, and 100% juice

### Perceived measures of community and household food resources

Individual evaluations of community food resources, stores where most of the groceries are purchased, and household food resources are presented in Table 3. Seniors believed community food resources were limited in variety of foods that can be purchased (32%), presence of few stores or supermarkets (59.6%), and high food prices (79.5%). Participants identified concerns with variety (10%), freshness (13.1%), or price (45.5%) of fruits and vegetables in the food store where most of their groceries were purchased. Almost 14% of respondents indicated that household food supplies in the month prior to the BVHA did not last and there was not enough money to buy more; 13% could not afford to eat balanced meals. Further, 48 (8.3%) respondents reported they had to cut the size of meals or skip meals in the past 12 months because there wasn't enough money to buy food.

**Table 3:** Perceptions of Rural Seniors on Adequacy of Community and Household Food Resources (*n *= 582)

	% (*n*)
***Community food resources***	
Little variety of foods that can be purchased	32.0 (186)
Few grocery stores or supermarkets	59.6 (347)
Food prices are high	79.5 (463)
***Food store where most groceries purchased***	
Variety of fruits and vegetables is fair/poor	10.0 (58)
Freshness of fruits and vegetables is fair/poor	13.1 (76)
Price of fruits and vegetables is fair/poor	45.5 (265)
***Household food resources***	
Food bought last month didn't last and we didn't have enough money to buy more	13.9 (81)
In the last month, we couldn't afford to eat balanced meals	13.1 (76)
In the past 12 months, we had to cut size of our meals or skip meals because there wasn't enough money to buy food	8.3 (48)

### Multivariable regression models of fruit and vegetable intake

Bivariate correlations with fruit and vegetable intake indicated poverty status, population density, and neighborhood deprivation were not significantly correlated with individual or combined fruit and vegetable intake (lowest *p *= 0.21). The remaining variables were entered in the first regression, which eliminated all variables with the exception of the following: individual characteristics (live alone, gender, and age) and food resources (food not last in the past month, few grocery stores or supermarkets in the community, and fair or poor variety of fruit and vegetables at the store where most of the household groceries were purchased). Heteroskedasticity and kurtosis of pertinent measures were determined to be within acceptable limits for the use of multivariable linear regression models. Tables 4, 5, 6, and 7 describe the association of individual characteristics, community and household food resources, and network distance to food stores with daily fruit and vegetable intake among rural seniors.

**Table 4:** Association of sample characteristics, community and household food resources, and network distance to food stores with fruit intake among 582 rural seniors, using multivariable linear regression models

	Model: Supermarket		Model 2: Fresh Fruit		Model 3: Fresh and Processed Fruit	
	**Coef (SE)^a^**	***P***	**Coef (SE)^a^**	***P***	**Coef (SE)^a^**	***P***

***Individual characteristics***						
Live alone	-0.171 (0.087)	0.050	-0.158 (0.087)	0.070	-0.170 (0.087)	0.051
Female	0.288 (0.084)	0.001	0.301 (0.084)	0.000	0.285 (0.084)	0.001
Age, y	0.028 (0.006)	0.000	0.028 (0.006)	0.000	0.029 (0.006)	0.000
***Food Resources***						
Food not last	-0.473 (0.108)	0.000	-0.464 (0.109)	0.000	-0.460 (0.108)	0.000
Few grocery stores	-0.073 (0.082)	0.371	-0.093 (0.081)	0.253	-0.098 (0.081)	0.227
Fruit/vegetable variety	-0.270 (0.112)	0.016	-0.281 (0.112)	0.012	-0.276 (0.110)	0.012
***Distance to nearest food store***						
Supermarket^b^	-0.012 (0.004)	0.003				
Fresh fruit^c^			-0.013 (0.007)	0.067		
Fresh and processed fruit^d^					-0.027 (0.009)	0.003

R^2^	0.111		0.105		0.112	
*P*	<0.0001		<0.0001		<0.0001	

^a ^SE = White-corrected standard errors

^b ^Network distance in miles from participant's residence to nearest supermarket

^c ^Network distance in miles from participant's residence to nearest food store with a good selection of fresh fruit

^d ^Network distance in miles from participant's residence to nearest food store with a good selection of fresh and processed (canned, frozen, and 100% juice) fruit

**Table 5:** Association of sample characteristics, community and household food resources, and network distance to food stores with vegetable intake among 582 rural seniors, using multivariable linear regression models

	Model 1: Supermarket		Model 2: Fresh Vegetables		Model 3: Fresh and Processed Vegetables	
	**Coef (SE)^a^**	***P***	**Coef (SE)^a^**	***P***	**Coef (SE)^a^**	***P***

***Individual characteristics***						
Live alone	-0.373 (0.086)	0.000	-0.362 (0.086)	0.000	-0.370 (0.086)	0.000
Female	0.206 (0.082)	0.013	0.217 (0.082)	0.008	0.210 (0.082)	0.011
Age, y	0.017 (0.005)	0.003	0.016 (0.005)	0.003	0.017 (0.005)	0.002
***Food Resources***						
Food not last	-0.495 (0.112)	0.000)	-0.486 (0.113)	0.000	-0.485 (0.113)	0.000
Few grocery stores	-0.226 (0.077)	0.004	-0.242 (0.077)	0.002	-0.240 (0.077)	0.002
Fruit/vegetable variety	0.128 (0.119)	0.281	-0.134 (0.119)	0.261	0.133 (0.119)	0.262
***Distance to nearest food store***						
Supermarket^b^	-0.008 (0.004)	0.033				
Fresh vegetables^c^			-0.007 (0.006)	0.267		
Fresh and processed vegetables^d^					-0.015 (0.009)	0.116

R^2^	0.125		0.119		0.121	
*P*	<0.0001		<0.0001		<0.0001	

^a ^SE = White-corrected standard errors

^b ^Network distance in miles from participant's residence to nearest supermarket

^c ^Network distance in miles from participant's residence to nearest food store with a good selection of fresh vegetables

^d ^Network distance in miles from participant's residence to nearest food store with a good selection of fresh and processed (canned, frozen, and 100% juice) vegetables

**Table 6:** Association of sample characteristics, community and household food resources, and network distance to fruit with combined fruit and vegetable intake among 582 rural seniors, using multivariable linear regression models

	Model 1: Supermarket		Model 2: Fresh Fruit		Model 3: Fresh and Processed Fruit	
	**Coef (SE)^a^**	***P***	**Coef (SE)^a^**	***P***	**Coef (SE)^a^**	***P***

***Individual characteristics***						
Live alone	-0.544 (0.150)	0.000	-0.517 (0.149)	0.001	-0.539 (0.149)	0.000
Female	0.494 (0.138)	0.000	0.521 (0.138)	0.000	0.491 (0.138)	0.000
Age, y	0.045 (0.009)	0.000	0.045 (0.009)	0.000	0.045 (0.009)	0.000
***Food Resources***						
Food not last	-0.968 (0.182)	0.000	-0.947 (0.184)	0.000	-0.945 (0.182)	0.000
Few grocery stores	-0.299 (0.132)	0.024	-0.332 (0.132)	0.012	-0.341 (0.131)	0.010
Fruit/vegetable variety	-0.399 (0.197)	0.043	-0.412 (0.196)	0.036	-0.407 (0.193)	0.036
***Distance to nearest food store***						
Supermarket^b^	-0.020 (0.006)	0.002				
Fresh fruit^c^			-0.017 (0.012)	0.153		
Fresh and processed fruit^d^					-0.043 (0.015)	0.005

R^2^	0.152		0.142		0.151	
*P*	<0.0001		<0.0001		<0.0001	

^a ^SE = White-corrected standard errors

^b ^Network distance in miles from participant's residence to nearest supermarket

^c ^Network distance in miles from participant's residence to nearest food store with a good selection of fresh fruit

^d ^Network distance in miles from participant's residence to nearest food store with a good selection of fresh and processed (canned, frozen, and 100% juice) fruit

**Table 7:** Association of sample characteristics, community and household food resources, and network distance to vegetables with combined fruit and vegetable intake among 582 rural seniors, using multivariable linear regression models

	Model 1: Supermarket		Model 2: Fresh Vegetables		Model 3: Fresh and Processed Vegetables	
	**Coef (SE)^a^**	***P***	**Coef (SE)^a^**	***P***	**Coef (SE)^a^**	***P***

***Individual characteristics***						
Live alone	-0.544 (0.150)	0.000	-0.521 (0.149)	0.000	-0.549 (0.149)	0.000
Female	0.494 (0.138)	0.000	0.518 (0.138)	0.000	0.494 (0.138)	0.000
Age, y	0.045 (0.009)	0.000	0.044 (0.009)	0.000	0.043 (0.009)	0.000
***Food Resources***						
Food not last	-0.968 (0.182)	0.000	-0.950 (0.184)	0.000	-0.947 (0.183)	0.000
Few grocery stores	-0.299 (0.132)	0.024	-0.340 (0.131)	0.010	-0.335 (0.132)	0.011
Fruit/vegetable variety	-0.399 (0.197)	0.043	-0.416 (0.197)	0.035	-0.415 (0.194)	0.033
***Distance to nearest food store***						
Supermarket^b^	-0.020 (0.006)	0.002				
Fresh vegetable^c^			-0.021 (0.011)	0.054		
Fresh and processed vegetable^d^					-0.046 (0.016)	0.004

R^2^	0.152		0.148		0.152	
*P*	<0.0001		<0.0001		<0.0001	

^a ^SE = White-corrected standard errors

^b ^Network distance in miles from participant's residence to nearest supermarket

^c ^Network distance in miles from participant's residence to nearest food store with a good selection of fresh vegetables

^d ^Network distance in miles from participant's residence to nearest food store with a good selection of fresh and processed (canned, frozen, and 100% juice) vegetables

Lower fruit intake (Table 4) among rural seniors was associated with living a greater distance to the nearest supermarket or food store with a good selection of fresh and processed fruit, controlling for individual characteristics and food resources. A 0.012 decrease in daily servings of fruit was observed for each mile to the nearest supermarket and 0.027 decrease for each mile to the nearest store with a good selection of fresh and processed fruit. Regardless of model, participants who resided in households where food purchased in the previous month did not last or who shopped in food stores where they considered the variety of fruits and vegetables as fair or poor consumed fewer servings of fruit. Increased age and female gender were associated with increased intake.

Lower vegetable intake (Table 5) was associated with living a greater distance from the nearest supermarket (0.008 decrease in daily servings of vegetables for each mile to the nearest supermarket); neither distance to nearest food store with a good selection of fresh vegetables nor good selection of fresh and processed vegetables was significantly correlated with vegetable intake. Lower vegetable intake was associated with food not lasting in the previous month, belief there were few grocery stores or supermarkets in their community, and living alone. Tables 6, 7 show the association of individual characteristics, food resources, and distance to food stores with combined intake of fruit and vegetables. As shown in table 6 and 7, increased distance to the nearest supermarket, increased distance to the nearest food store with a good variety of fresh and processed fruit, or increased distance to the nearest food store with a good selection of fresh and processed vegetables were associated with lower intake of combined fruit and vegetables. Each mile in distance to a supermarket was associated with a 0.02 reduction in daily servings; and each mile to fresh and processed fruit or vegetables was associated with 0.043 and 0.046 reduction in daily servings of fruit and vegetables. In addition, decreased number of combined servings was associated with living alone, food not lasting, and believing that there were few grocery stores or supermarkets in the community or that the variety of fruits and vegetables was fair or poor. Increased age and being female were associated with increased intake.

## Discussion

The importance of fruit and vegetable consumption to health has been well established [7,66-68]. Considering the importance of geographic access to retail food resources, few studies have focused on access to fruit and vegetables in rural areas [16,50,65]; even fewer examined the relationship between food access and fruit and vegetable consumption among seniors, regardless of location [44]. This study extends our understanding of spatial challenges to nutritional health faced by seniors in a large rural area lacking public transportation. This is the first study, to our knowledge, that examines the relationship between spatial access to food stores, especially food stores providing a good variety of fresh and processed (canned, frozen, and 100% juice) fruit and vegetables, and daily intake of fruit, vegetables, and combined fruit and vegetables among rural seniors. Our analyses revealed that increased distance to the nearest supermarket, food store with a good variety of fresh and processed fruit, or food store with a good variety of fresh and processed vegetables was associated with decreased daily consumption of fruit, vegetables, and combined fruit and vegetables, after controlling for the influence of individual characteristics and perceptions of community and home food resources. The findings suggest that interventions designed to increase fruit and vegetable consumption among rural seniors should consider strategies to ameliorate differential access to healthy food due to food store distance. The implications of the findings regarding distance measures, however, should be tempered with the magnitude of the regression coefficients. For participants at the 75^th ^percentile for distance to the nearest supermarket, distance was associated with less than one-fifth serving of fruit, one-tenth of a serving of vegetables, and more than one-fourth of a serving of combined fruit and vegetables. At the same percentile for distance to the nearest food store with a good selection of fresh and processed fruit, this equated to one-fifth of a serving of combined fruit and vegetables. This equated to almost one-third of a serving for the 75^th ^percentile of distance to the nearest food store with a good selection of fresh and processed vegetables.

Although the food environment experienced by rural seniors is different from the food environment experienced by seniors in high-population-density urban and suburban areas [53], this study found a similar negative influence on fruit and vegetable consumption by retail store distance as that reported among 257 seniors in Brooklyn, NY [44]. There were some noticeable differences between the NY sample and our rural sample. First, the mean intake of combined fruit and vegetables was lower among the present study of rural seniors (3.58 in the rural sample compared with approximately 5 servings in the Brooklyn sample). The mean difference in combined fruit and vegetable intake may be explained by the instruments used to estimate intake. The present study used a two-item screener that separately asked each respondent to indicate the number of servings usually consumed each day of fruit or vegetables. The intake among Brooklyn seniors was estimated using the National Cancer Institute Fruit and Vegetable Screener, which separately asks the frequency over the last month and the amount consumed each time for 100% juice, fruit, lettuce salad, French fries or fried potatoes, other white potatoes, cooked dried beans, other vegetables, tomato sauce, vegetable soup, and other mixtures that include vegetables [44,69]. Separate questions on lettuce salads, French fried potatoes, tomato sauce, vegetable soups, and mixtures that included vegetables may be responsible for the larger estimated intake in the Brooklyn sample. Second, mean distance to the primary store for grocery purchase was much farther in this rural sample (14.8 miles vs. 0.8 miles). Finally, a higher percentage of rural seniors were married, a smaller percentage lived alone, and men comprised a larger proportion of the rural sample. The Brooklyn sample was recruited from 10 senior centers, where participants are more likely female or live alone [70]. In contrast, the present rural sample was randomly recruited through random-digit dialing and may better represent older adults in the rural areas. In addition, perceptions of community and household food resources, which were not included in the urban regression analyses, were consistently and negatively associated with fruit and vegetable intake. Others have also found that the perception of the community food environment influences the food chosen for the household [48,71]. Furthermore rural residents are more likely to believe they had restricted access to food resources, higher food costs, and poor quality and variety [53,72].

Several additional findings warrant further mention: 1) the distance to the nearest food store with a good selection of fruit or vegetables decreased when fruit or vegetables included canned, frozen, and 100% juice types in addition to fresh; 2) perception of fair or poor variety of fruit and vegetables in the store where most of the groceries were purchased was associated with decreased daily fruit intake (greater than one-quarter serving), but not vegetable intake; 3) perceptions there were few grocery stores or supermarkets in their community was associated with decreased intake of vegetables and not fruit; 4) neighborhood socioeconomic deprivation and population density were not associated with fruit and vegetable intake; 5) negative perceptions of community food resources were consistently associated with decreased intake of combined fruit and vegetables; and 6) the magnitude of association with decreased intake of fruit, vegetables, and combined fruit and vegetables was largest for limited household food resources; that is, food purchased in the past month not lasting and no money available to purchase more food. Generally, studies have shown that neighborhood access to a supermarket influences individual fruit and vegetable consumption [8,33,73-77]. However, the results may be different when we consider neighborhood characteristics, such as socioeconomics, racial mix, or population density. Although our prior work found that high deprivation rural neighborhoods (CBG) had better relative potential access to a supermarket than other neighborhoods and neighborhoods of low population density had worse access [51], this study found no statistically significant relationship (unadjusted or adjusted) of either neighborhood deprivation or population density to fruit and vegetable intake. Our finding of no association between neighborhood deprivation and fruit and vegetable intake is similar to the findings in a couple of non-U.S. studies [74,75] and dissimilar to a U.S. study using NHANES data [73]. Neither neighborhood socioeconomic deprivation nor population density was included in the recent study of NY seniors [44].

This study linked two contemporaneous datasets (BVFEP and BVHA) with the 2000 U.S. Census. The BVFEP food store data in this study were originally collected using ground-truthed methods that involved direct observation of all food stores and food service places in all six rural counties, on-site collection of locational points using mobile Global Positioning System, and on-site collection of presence of fresh, canned, frozen, and 100% juice fruit and vegetables. The ground-truthed method provided more accurate information than utilization of publicly available food stores lists [51]. This approach responded to methodological challenges that have been identified in measuring potential access to food stores in rural areas [78]. Considering that supermarkets and grocery stores are no longer the only shopping opportunities for fruit and vegetables, BVFEP data recognized the emergence of new and changing food store formats [65]. Not only was this study able to calculate the network distance from the residence of all rural seniors in the BVHA to the nearest supermarket, but also to the nearest food store offering a good selection of fresh fruit, fresh vegetables, fresh and processed fruit, or fresh and processed vegetables [55,65].

This study has several limitations. Daily consumption of fruit and vegetables was estimated through a self-reported, self-administered two-item survey, which is subject to measurement error. Unlike the Brooklyn study, the identification of participant's primary grocery destination was not available [44]. As a result, our distance measures describe potential spatial access to the nearest locations. Although the respondents were representative of the population distribution geographically and persons with a household income below the poverty threshold, women were overrepresented and racial/ethnic minorities (African Americans and Hispanics) and individuals with limited education (completed less than 9^th ^grade) were underrepresented in the survey sample [62]. Another limitation is lack of data on social support. Additionally, the cross-sectional nature of the data prevents an examination of causality in fruit and vegetable intake. Confirmation of these findings in other rural senior populations is needed.

Despite these limitations, the data presented suggest that distance to the nearest supermarket or food store, regardless of type, with a good selection of fresh and processed fruit or vegetables was associated with daily consumption of fruit, vegetables, and combined fruit and vegetables. For rural seniors, increased distance to food stores was associated with decreased fruit and vegetable intake. Further, inadequate household food resources and perceptions of fair or poor community food resources were also associated with lower intake of fruit and vegetables among rural seniors. This is particularly important, given that there has been limited attention to environmental factors that may influence food choice and dietary intake among rural seniors. As important as easy access to community food sources are to a healthy diet, rural seniors are particularly disadvantaged. For rural seniors, many of whom have to watch their fixed income, the changing grocery store environment translates into a lack of choice in food store destination where they shop, limited selection, and higher prices [79,80]. Having to shop outside the area where they live, rural seniors face challenges with private transportation or increased dependency on others [53].

## Conclusion

Rural areas have reported disadvantages when it comes to availability, accessibility, and adequacy of health and social services and healthy foods, which particularly affects seniors [57,81]. This study goes beyond prior studies by examining access to food stores and availability and variety of fresh and processed fruit and vegetables by rural seniors. Seniors who reside in rural areas may be influenced to a large extent by the demands placed on individuals by adequacy of home, neighborhood, and community resources. Rural seniors' nutritional health may face greater challenges due to limited resources, difficulties with transportation, and greater distance to food resources. Indeed, many rural seniors who do not drive must rely on family, friends, and neighbors for transportation [81]. The findings of this study show that rural seniors consume few daily servings of fruit and vegetables. Rural seniors who live alone, have inadequate household and community food resources, or live a greater distance from the nearest supermarket or food store offering a good selection of fresh and processed fruit or vegetables are most at risk for low fruit and vegetable intake.

Thus, greater attention must be directed toward the availability and utilization of food resources in rural areas. To foster creative and effective community-based approaches to meeting dietary needs, prospective research needs to be conducted, which identifies the household, neighborhood, and community barriers and facilitators to healthful food choices. Additional research is needed to better understand older consumers and how characteristics of the home and community food environment in rural areas serve as barriers and facilitators for healthful eating. Interventions targeting the prevention and management of nutrition-related health conditions, especially for rural seniors, should understand the context in which rural seniors live and shop, and recognize the influence of access and availability to healthy food on an individual's ability to initiate and maintain a healthy nutritional lifestyle. Educational interventions need to emphasize the availability of healthy foods in non-conventional locations such as convenience and dollar stores. Furthermore, considering the importance of all vegetables and all fruits in this study, they should also focus on the beneficial nutritional characteristics of frozen and canned fruits and vegetables.

It is difficult to initiate or maintain healthful eating habits without access to healthful foods. Large numbers of an increasingly diverse older population are living in rural areas; many of whom face the burden of disease, increased economic constraints, and greater spatial inequality for access to healthful food. Indeed, the preparation for policy change to strengthen food assistance programs or program delivery activities, or interventions to improve nutritional health of this growing population should include an understanding of the community - where people live and where they shop for food [82].

## Competing interests

The authors declare that they have no competing interests.

## Authors' contributions

JRS developed the original idea for the study. JRS wrote the first draft of the paper. JRS, CMJ, and WRD read and approved the final manuscript.

## Pre-publication history

The pre-publication history for this paper can be accessed here:

http://www.biomedcentral.com/1471-2318/10/32/prepub
